# Draft Genome Sequences of *Proteus mirabilis* K1609 and K670: A Model Strains for Territoriality Examination

**DOI:** 10.1007/s00284-018-1598-6

**Published:** 2018-11-17

**Authors:** Dawid Gmiter, Grzegorz Czerwonka, Justyna Malgorzata Drewnowska, Izabela Swiecicka, Wieslaw Kaca

**Affiliations:** 1 Department of Microbiology, Jan Kochanowski University, 15 Swietokrzyska Street, 25-406 Kielce, Poland; 2 Departament of Microbiology, Institute of Biology, University of Bialystok, 1J Ciolkowskiego Street, 15-245 Bialystok, Poland; 3 Laboratory of Applied Microbiology, University of Bialystok, 1J Ciolkowskiego Street, 15-245 Bialystok, Poland

## Abstract

*Proteus mirabilis* is a pathogenic Gram-negative bacterium characterized by its ability to swarm across surfaces, which frequently leads to colonization of the urinary tract and causes severe infections. *P. mirabilis* strains are also well known from their self-recognition phenomenon, referred to as Dienes phenomenon. In this study, we present novel aspect of self-recognition, which is a hierarchy in terms of strains territoriality. We report the draft genome sequences of *P. mirabilis* K1609 and K670 strains exhibiting the strongest and the weakest territoriality, respectively. Our results indicated that K1609 is closely related to strain BB2000, a model system for self-recognition, comparing with the K670. We annotated genes associated with recognition of kin and swarming initiation control and indicated polymorphisms by which observed differences in territoriality might results from. The phenotypic and genomic features of both strains reveal their application as a model organisms for studying not only the mechanisms of kin-recognition but also strains territoriality, thus providing new approach to the phenomenon. Availability of these genome sequences may facilitate understanding of the interactions between *P. mirabilis* strains.

## Introduction

*Proteus mirabilis* is a Gram-negative urinary tract pathogen that exhibits remarkable ability to swarm over the solid surface. Swarming motility is a complex social behavior requiring cell to cell communication, and possible virulence factor allowing *P. mirabilis* to gain access to the bladder by migration along the external surfaces of the catheter [1]. *P. mirabilis* is also well known from its territorial behavior, which manifests in form of the demarcation line formation at the edge of approaching swarms. The phenomenon, referred to as Dienes phenomenon, is known for over a five decades [2]. Demarcation line occurs between non-kin strains and the process is governed by the action of type VI secretion system (TVISS) exporting proteins that determinate strains kin recognition [3, 4]. The previous studies indicated the role of *idsABCDEF* and *idrABCDE* [4] as well as primary *hpc-vgrG* effector (*pef)* operons [3, 5] in the phenomenon; however, the exact mechanism is poorly understood.

To date, only *P. mirabilis* HI4320 and *P. mirabilis* BB2000 were completely sequenced [6, 7] as an examples of strains employed in self-recognition and competition examination [5, 8]. The BB2000 strain was first in which the self-recognition genes were identified [9]. Here, we present novel aspect of self-recognition in *P. mirabilis*, which is a hierarchy in terms of strains territoriality, and report genome sequences of *P. mirabilis* K1609 and K670 strains. Both strains have been chosen because of their remarkably differences in territorial advantages on solid surface. Based on phenotypic and genomic differences, we proposed these strains as a model organisms for territoriality examination. Our two-strains system is unique comparing to the previously used. It allows for a thorough investigation focused on the mechanisms of territoriality among *P. mirabilis*, which contributes to a better understanding of the phenomenon.

## Materials and Methods

### Strains, Genome Sequences, and Territoriality Assay

The five *P. mirabilis* strains used in this study are presented in Table 1. Study included three clinical isolates from the Holly Cross Cancer Center in Kielce and two laboratory strains obtained from the Czech National Collection of Type Cultures in Prague, Czech Republic. Following the matrix laser desorption ionization-time of flight mass spectrometry (MALDI-TOF MS) spectrum analysis, strains were deposited in Polish Collection of Microorganisms of the Ludwik Hirszfeld Institute of Immunology and Experimental Therapy, Polish Academy of Science, Wroclaw. Strains were maintained in Lysogenic broth (LB) (Biocorp, Poland) supplemented with 8% DMSO at the temperature of − 80 °C. Used genome sequences of *P. mirabilis* strains are presented in Table 2. The LB was used for culturing, and LB plates with the 1.5% agar (Biocorp, Poland) were used for swarming experiments—swarming agar. For the territoriality examination, the 2.5 µl of strains suspensions (1:100 dilution of overnight culture) were spotted in opposition onto the swarming agar plates and allowed to swarm for 18 h at 37 °C.

**Table 1:** Strains used in the study

Strain	Reference and source	#PCM*
K1609	This study; Holly Cross Cancer Center in Kielce, Poland	2877
K670	[10]; Holly Cross Cancer Center in Kielce, Poland	2871
K12796	This study; Holly Cross Cancer Center in Kielce, Poland	2866
PrK 34/57	[10]; Czech National Collection of Type Cultures in Prague, Czech Republic	2874
PrK 61/57	[10]; Czech National Collection of Type Cultures in Prague, Czech Republic	2875

*#*PCM* Deposition number in Polish Collection of Microorganisms

**Table 2:** Complete and draft genome sequences of *Proteus mirabilis* strains used in this study

Strain	Accession number	Genome size (bp)	Data type	Reference
BB2000	CP004022	3,846,754	Complete	[7]
HI4320	AM942759	4,063,606	Complete	[6]
GN2	CP026581	4,012,640	Complete	[11]
1230_SSON	NZ_JVXV01000000	3,923,692	WGS	[12]
AOUC-001	CP015347	4,272,433	Complete	[13]
AR_0029	CP029725	3,980,098	Complete	
AR_0155	CP021694	4,372,742	Complete	
AR_0159	CP021550	4,055,152	Complete	
AR379	CP029133	4,219,380	Complete	[14]
ATCC 7002	NZ_JOVJ00000000	3,992,612	WGS	[15]
BC11-24	CP026571	4,021,165	Complete	[16]
CYPM1	CP012674	3,793,000	Complete	
PM_125	NZ_LWUL00000000	3,955,474	WGS	[17]
PM_178	NZ_LWUM00000000	3,969,065	WGS	[17]
Pr2921	LGTA00000000	3,924,499	WGS	[18]
T18	CP017085	4,131,426	Complete	
WGLW4	NZ_AMGU00000000	3,920,397	WGS	
ATCC 29906	NZ_ACLE01000000	3,975,048	WGS	

### Sequencing

Genomic DNA was isolated using the QIAamp DNA Micro Kit (Qiagen GmbH, Hilden, Germany) according to the manufacturer’s procedure with a protocol for Gram-negative bacteria. Final elution was performed with nuclease-free water. DNA quality was assessed using a NanoDrop 2000 Spectrophotometer (Thermo Fisher Scientific, Wilmington, USA). The quantity was measured using both the Qubit 2.0 Fluorometer with Qubit dSDNA HS Assay Kit (Invitrogen, Thermo Fisher Scientific, Wilmington, USA) and the 2200 TapeStation Instrument with Genomic DNA ScreenTape Assay (Agilent Technologies Inc., St Clara, CA, USA). Libraries were prepared using the Nextera XT kit (Illumina Inc., San Diego, CA, USA) according to the manufacturer’s protocol and quantified by capillary electrophoresis applying the Agilent High Sensitivity D5000 ScreenTape System (Agilent Technologies Inc.). Libraries were sequenced on the MiSeq machine (Illumina) using v2 reagents with 2 × 250 bp paired-end reads. Consequently, 90.2 and 82.4% of bases of sequencing reads had quality scores ≥ Q30 for K1609 and K670, respectively. De novo genome assembly was performed using CLC Genomic Workbench v5 (Qiagen). Plasmid DNA was isolated using AccuPrep Plasmid Mini Extraction Kit (Bioneer Company, Daejeon, South Korea) according to the manufacturer’s procedure.

### Bioinformatics

Genome sequences were functionally annotated by Rapid Annotation Subsystems Technology (RAST) server [19] using the ClassicRAST annotation scheme, FIGfams version 90, automatic error correction, and automatic frame shift correction. The genetic relationships of strains were presented using average nucleotide identity (ANI) calculator (http://enve-omics.ce.gatech.edu/ani/index) [20]. The phylogenomic tree was obtained using T-Rex (http://www.trex.uqam.ca/) employing Neighbor-joining method [21]. The genes associated with strains territoriality were annotated manually using BLAST 2.8.0 [22]. For genomes’ visual comparison Mauve software was used [23].

## Results and Discussion

### Strains Territoriality

Two *P. mirabilis* strains inoculated on the agar plate start to migrate toward each other. When migrating swarms meet, the formation of Dienes line occurs, if both belong to different Dienes compatibility groups [24]. Our observation allowed to point out that strains display different ability to space occupying. Therefore, we referred territoriality among *P. mirabilis* as ability of two non-kin swarms to occupy the surface of agar plate in presence of each other.

**Fig. 1:**
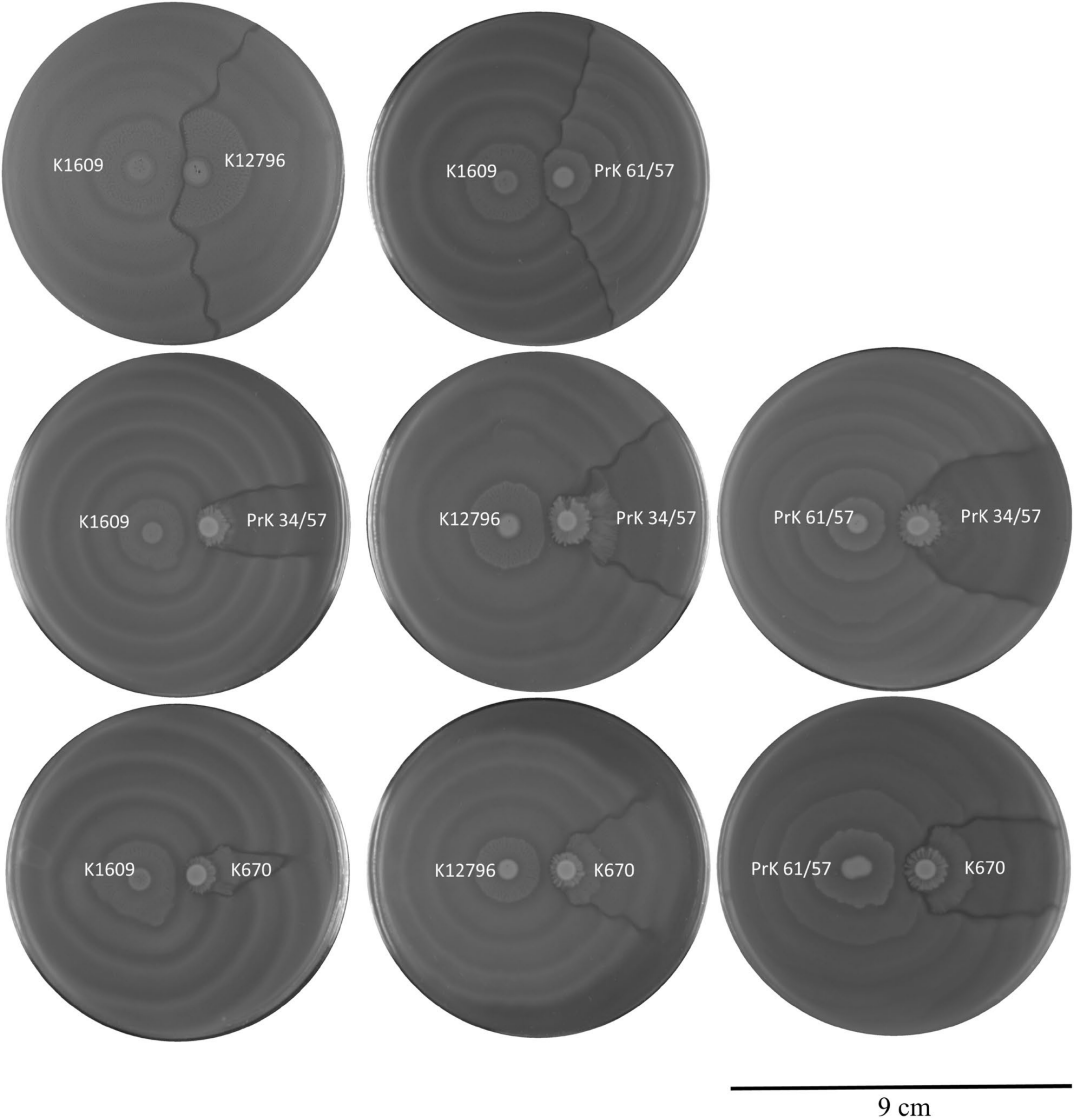
Territoriality of studied *Proteus mirabilis* strains. *P. mirabilis* strains exhibit hierarchy in terms of their territorial advantages. Territoriality is defined as the area of surface occupied by particular swarm in presence of non-kin competitor. Strains K1609 and K670 demonstrate the strongest and the weakest territoriality among studied strains, respectively

All five strains submitted to territoriality assay formed Dienes line with each other. The territorial behavior of strains is presented in Fig. 1. In all used combinations, the K1609 tended to occupy larger area of plate comparing to second strain—we defined this as strong territorial advantages. Isolate K12796 and laboratory strain PrK 61/57 exhibited moderate territoriality. The K12796 and PrK 61/57 growth was restricted by K1609 to some extent. Territory of strains K670 and PrK 34/57 was restricted at the highest level in the presence of other competitors so we define this as the weakest territorial advantages. The restriction effect was not so intense for PrK 34/57 comparing with the K670. Strain K1609 restricted the K670 growth at the highest level comparing to the restriction caused by the remaining strains. Thus, strains K1609 and K670 exhibit the strongest and the weakest territoriality, respectively.

Taking account the observation above, it could be stated that among used *P. mirabilis* strains the hierarchy in terms of territoriality occurs. To our best knowledge the results obtained in this study is the first report of such hierarchy in *P. mirabilis* territoriality. Previously, it was said that colonization of the plate is largely determined by the rate and initiation of swarming [24]. However, the mechanisms governing that process and its eventual biological importance remain unexplained.

### Genomes Characterization and Phylogenomic

Based on the observation above, K1609 and K670 were selected for DNA sequencing. The main features of strains genomes are presented in Table 3. Both strains possess quite similar genomes size and a number of predicted coding sequences. The distribution of subsystems in K1609 and K670 is presented in Table 4. Only in K1609 we observed the presence of one plasmid, which was not sequenced separately.

**Table 3:** Genomes assembly statistics

Attribute	Value	
	K1609	K670
Genome size (bp)	3,817,795	3,935,626
%GC	38.5	38.7
N50 (bp)	95,718	105,852
L50 (bp)	13	13
Number of contigs (with PEGs)	83	76
Number of subsystems	496	496
Number of coding sequences	3455	3568
Number of RNAs	78	82
Number of plasmids	1	0

**Table 4:** Subsystems distribution of *Proteus mirabilis* K1609 and K670 strains based on RAST annotation server

Subsystems	K1609	K670
Cofactors, vitamins, prosthetic groups, pigments	248	248
Cell wall and capsule	159	160
Virulence, disease, and defence	71	73
Potassium metabolism	25	25
Photosynthesis	0	0
Miscellaneous	43	43
Phages, prophages, transposable elements, plasmids	25	23
Membrane transport	182	180
Iron acquisition and metabolism	55	58
RNA metabolism	223	223
Nucleosides and nucleotides	98	97
Protein metabolism	269	275
Cell division and cell cycle	37	36
Motility and chemotaxis	57	57
Regulation and cell signaling	97	97
Secondary metabolism	4	4
DNA metabolism	104	96
Fatty acids, lipids, and isoprenoids	109	108
Nitrogen metabolism	26	26
Dormancy and sporulation	6	6
Respiration	150	151
Stress response	134	134
Metabolism of aromatic compounds	3	3
Amino acids and derivatives	381	366
Sulfur metabolism	36	17
Phosphorus metabolism	35	37
Carbohydrates	332	331

**Fig. 2:**
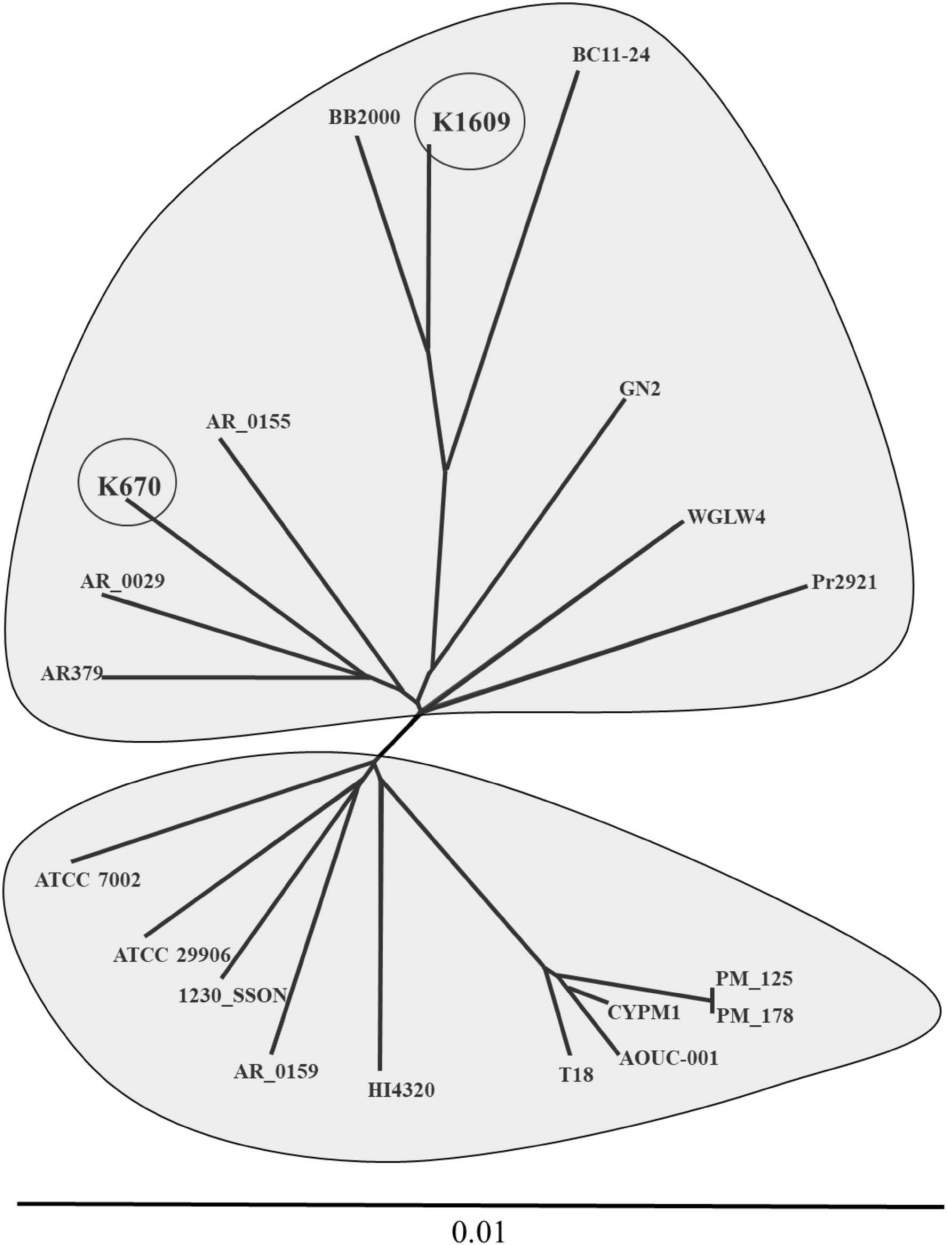
Neighbor-joining tree of *Proteus mirabilis* K1609 and K670 and closely related *P. mirabilis* strains based on whole-genome ANI distance matrix

**Fig. 3:**
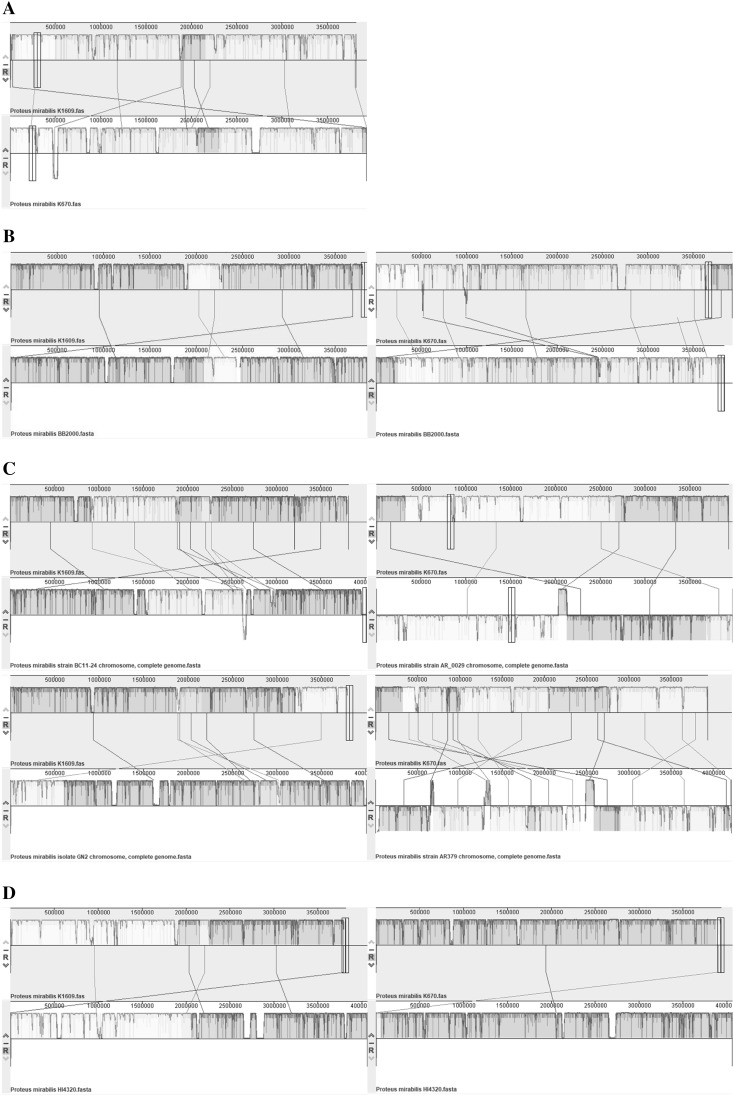
Mauve comparison of *Proteus mirabilis* genomes. Pair-wise Whole Genome Alignment of **a***P. mirabilis* strain K1609 against K670, **b** K1609 and K670 against BB2000, **c** K1609 against close relatives BC11-24 and GN2 and K670 against close relatives AR_0029 and AR379, **d** K1609 and K670 against HI4320

Phylogenomic analysis based on whole-genome ANI distance matrix revealed that *P. mirabilis* genomes clustering into two major clades (Fig. 2). We referred these clades as BB2000-like and HI4320-like. Both our studied strains are located in BB2000-like group; however, K1609 is closely related to BB2000 comparing with the K670. The divergence into two clades represented by BB2000 and HI4320 was previously shown by the phylogenetic analysis using 16S rRNA gene [25]. Genome of K1609 clusters with the BB2000, BC11–24 and GN2, meanwhile K670 clusters with the AR_0029, AR379 and AR_0155. Previously it was shown that BB2000 and HI4320 strains exhibit genetic variations corresponding to self-recognition differences [7], which is in line with our observation. Visual comparison of K1609 and K670 with BB2000, HI4320 and their closest relatives is presented in Fig. 3.

### Annotation of Genes Potentially Involved in Strains Territoriality

Our hypothesis assumed that strains exhibiting strong territoriality start migration earlier, which allows to colonize of larger area of plate than it is possible for weak competitors at the same time. After the non-kin swarms contact, spreading of weaker competitor is restricted through the self-recognition mechanisms [3, 4]. At this point, the crucial factors involved in strains territoriality seem to be the migration initiation control and self-recognition mechanisms.

We decided to annotate genes associated with the self-recognition in *P. mirabilis*. The RAST annotation predicted in K1609 and K670 genomes the presence of genes encoding Hpc an VgrG proteins, which are the structural elements of TVISS machinery [26]. Using BLAST comparative analysis, we confirmed the presence of putative TVISS gene locus in both strains. This putative TVISS locus is highly conserved (99% of homology) with the previously described in BB2000 and HI4320 strains [3, 4].

**Fig. 4:**
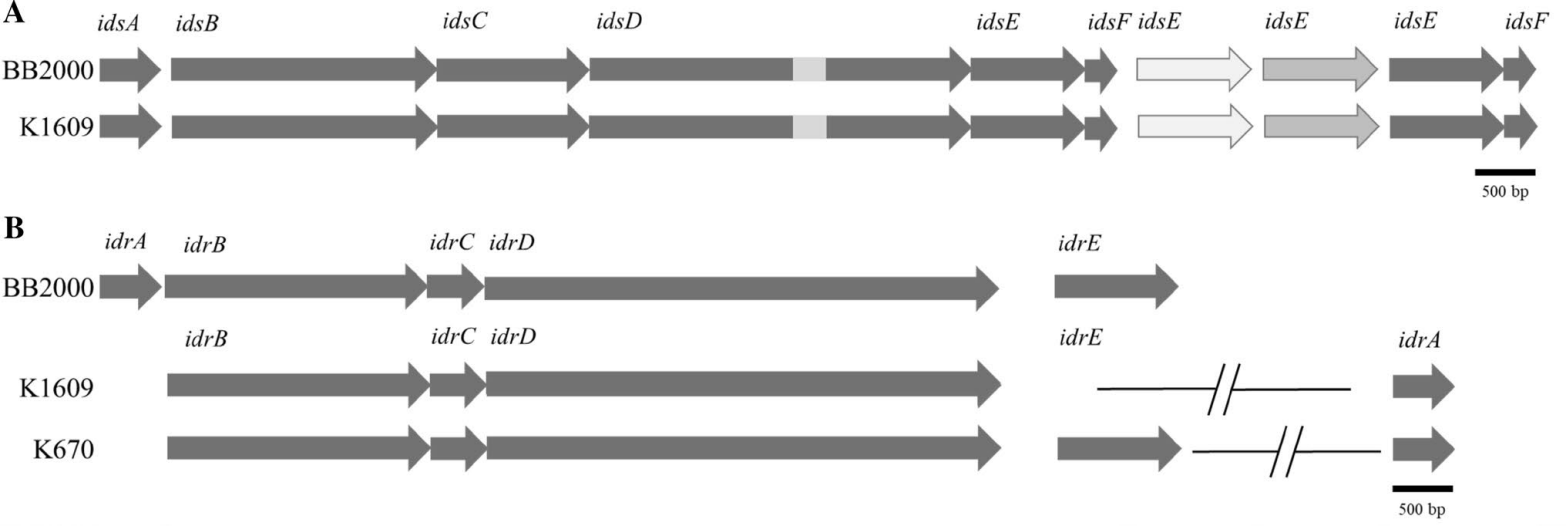
Comparison of **a***idsABCDEF* and **b***idrABCDE* genes between *Proteus mirabilis* BB2000, K1609 and K670 strains. For Panel **a** gray scale indicates the level of genes homology. The region of low similarity in *idsD* between BB2000 and K1609 is marked with light gray. For Panel **b** slanted lines indicate a break in the genomic regions, corresponding to approximately 2 Mbp

As the *P. mirabilis* BB2000 was the first strain in which self-recognition genes were identified [9], we decided to annotate *ids* and *idr* operons in K1609 and K670 through manually comparison using BLAST algorithm. Comparison of *ids* and *idr* genes between BB2000, K1609, and K670 is presented in Fig. 4. The BLAST analysis revealed that K670 strain lacks the *ids* operon, meanwhile these genes are present in the genome of K1609. The lack of *ids* operon in K670 is intrigued considering the role of this operon in *P. mirabilis* self-recognition [27]. However, our screening additionally revealed absence of *ids* operon in complete genomes of AR_0195 and 1230-SSON strains and draft genome of WLGW4. This observation is interesting considering the phylogenomic analysis presented in Fig. 2. It could be seen that only WGLW4 belongs to the same clade as K670 in opposition to AR_0195 and 1230-SSON, which are located in HI4320-like clade.

We observed as well that *idsEF* genes are at least duplicated in K1609 strain, which in fact is not precedence. Additional copies of *isdEF* are also present in BB2000. Its role in self-recognition as an orphan genes is speculative [9]. The comparison analysis of BB2000 and K1609 strains shown 99–100% of homology between *idsABCDEF* genes. However, the *idsD* gene possesses a fragment of low homology between K1609 and BB2000 in the central part. Strains K1609 and BB2000 demonstrate homology in *ids* operon organization (Fig. 4a), which corresponds to their genomic similarities.

Both K1609 and K670 strains possess the *idrABCD* genes, whereas the *idrE* was found only in the strain K670. In both genomes *idrA* is located at a considerable distance from the *idrBCDE* cluster. The *idrD* gene from BB2000, potentially encoding the toxic protein [4], share 99% and 98% identity with K1609 and K670, respectively (Fig. 4b). Using BLAST we were not able to detect significant homology to gene encoding the PefD toxin of *pef* operon presented in HI4320 strain [5]. Differences in *ids* and *idr* operon between K1609 and K670 might be the molecular factor responsible for the strains recognition as non-kin.

The overexpression of *rsbA* gene contributes to the precocious phenotype in *P. mirabilis* that is characterized by defect in the temporal control of swarming migration. Such strains start swarming ca. 60 min. earlier [28]. After annotation, we observed differences in sequence of *rsbA* between K1609 and K670. In both strains, this polymorphism did not contribute to the amino acid sequence of RsbA protein, comparing to BB2000. Nevertheless, it cannot be rejected that these silent mutations do not contribute to the RsbA function most likely by a distorted balance of the protein folding process [29]. Next we identified a single point mutation in *rcsC* gene in K670 genome. The mutation results in serine presented in BB2000 and K1609 at 873 position substitution with the arginine. The mutation occurred in the region of receiver domain in RcsC protein [30]. The RcsB and RcsC are members of a two-component regulatory circuit controlling capsular synthesis, where RcsC is a histidine kinase and RcsB is its cognate response regulator. The *rcsB* and *rcsC* are located in *P. mirabilis* downstream the *rsbA*. It was shown that distribution of *rcsC* gene in *P. mirabilis* BB2000 results in similar precocious phenotype as in case of *rsbA* [28]. In both studied strains, we did not detect any missense mutation in *rcsB* gene.

## Conclusions

Within presented genome announcement, we report draft genome sequences of two *P. mirabilis* strains that exhibit differences in terms of territoriality advantages. We hypothesize the possible role of differences within *rscC* and self-recognition genes in swarming initiation control and recognition of kin, respectively. Our in silico analysis provided basic genomic insight that will serve for further examination of the self-recognition and territoriality in *P. mirabilis* K1609 and K670 model system. The *P. mirabilis* genome sequences obtained in this work were deposited at GenBank and are available under the Accession Numbers CP028522 and CP028356, for K1609 and K670, respectively.
